# Microglial cathepsin B is necessary for neuronal efferocytosis in zebrafish and mice during brain development

**DOI:** 10.1038/s41467-026-70350-1

**Published:** 2026-03-13

**Authors:** Nicholas J. Silva, Sarah R. Anderson, Supriya A. Mula, Caroline C. Escoubas, Haruna Nakajo, Anna V. Molofsky

**Affiliations:** 1 Department of Biology, San Francisco State University, San Francisco, CA USA; 2 Department of Psychiatry and Behavioral Sciences, Weill Institute for Neurosciences, San Francisco, CA USA; 3 Kavli Institute for Fundamental Neuroscience, University of California, San Francisco, CA USA

**Keywords:** Microglia, Neuroscience, Cell death in the nervous system

## Abstract

Half of all newborn neurons in the developing brain are removed via efferocytosis - the phagocytic clearance of apoptotic cells. Microglia are brain-resident professional phagocytes that play important roles in neural circuit development including as primary effectors of efferocytosis. While the mechanisms through which microglia recognize potential phagocytic cargo are widely studied, the lysosomal mechanisms that are necessary for efficient digestion are less well defined. Here we show that the lysosomal protease cathepsin B is enriched in microglia located in brain regions where neuronal turnover is high in both zebrafish and mouse. Genetic disruption of cathepsin B in zebrafish and mice had an accumulation of microglia containing undigested dead cells. Live imaging studies in zebrafish and in cultured mouse microglia revealed fewer phagocytic events and reduced overall phagocytosis. We also observed behavioral impairments in both models. These data reveal a role for microglial cathepsin B in vertebrate brain development.

## Abstract (web summary)

Microglia, the brain’s immune cells, are important for neuronal phagocytosis during brain development. Here the authors show that microglial lysosomal protease cathepsin B is required for efficient neuronal efferocytosis during brain development.

## Introduction

Microglia are the professional phagocytes of the brain and are essential participants in brain development^1–3^.They survey the brain parenchyma in an activity-dependent manner and can rapidly phagocytose portions of cells or entire cells during development, plasticity, and in disease^4,5^. Microglia are vastly outnumbered by neurons in the developing brain, yet they are highly efficient phagocytes, eliminating up to 30% of all cells produced in the cerebral cortex^6^. The vast majority of this cell removal occurs via efferocytosis, a phagocytic mechanism for engulfing apoptotic cells^7^. This is an energetically demanding process, particularly during development, when neuronal turnover is high. Therefore, defining the cellular mechanisms that affect efferocytosis by microglia is important to understand physiological brain development as well as pathogenesis of neurodevelopmental and neurodegenerative diseases.

Efferocytosis is a multistep process. Microglia are first recruited to the dying cell, then identify dead cell cargo via a series of “eat me” signals, including phosphatidylserine, which are present on the surface of apoptotic cells^7^. They then internalize the cargo into a phagosome which fuses with lysosomes to enable digestion within the phagolysosome^7–9^. This fusion event generates an acidified phagolysosome where hydrolytic enzymes degrade proteins, nucleic acids, and other cellular material^10^. The last stage of phagocytosis is resolution, during which the phagolysosome is compacted, its digestion products eliminated, and reusable elements including membranes recycled for subsequent rounds of phagocytosis^11^. Some pathways have been identified, including vacuolar ATPases that promote acidification^12^, Slc37a2, a glucose transporter that promotes compaction of the phagosome^13^, clathrin-dependent phagosome resolution^11^, and Type I interferon signaling, which is necessary for whole cell phagocytosis through as yet undefined mechanisms^14^. One clear outcome of these studies is that impairment at any stage of the digestion process can reduce the efficiency of phagocytosis, leading to a backup of undigested material. While many aspects of microglial efferocytosis have been extensively studied, the role of lysosomal digestive proteins and how they process phagocytic cargo are relatively less well understood.

Efficient digestion requires degradation of proteins in the phagolysosome. This process depends largely on cathepsins, the most abundant lysosomal proteases. There are three families of cathepsins, which include serine proteases (A and G), aspartic proteases (D and E) and cysteine proteases (B, C, F, H, L, O, S, V, X, and W)^15,16^. Cathepsin B is synthesized as a zymogen, an inactive enzyme that requires autocatalysis for full maturation within the lysosome. Under physiological states, cathepsin B is localized to the lysosome and phagosome, where it aids in proteolysis of engulfed proteins^15,16^. However, it can also be pathologic when released from the lysosome by physiological stress and in disease states^17^. Cathepsin B is highly upregulated in disease-associated microglia (DAMs) observed in aging and diseases, including in neurodegenerative diseases^18–20^. It is also physiologically expressed in microglia across vertebrates including mammals and zebrafish^21^. We previously identified cathepsin B as highly expressed in developing zebrafish microglia and particularly enriched in microglia known to engulf apoptotic corpses in the optic tectum^22^. Thus, while cathepsin B is predicted to be an important lysosomal protease in microglia, its effects on phagocytosis in physiologic brain development have not been examined.

Here, we studied the function of microglial cathepsin B in brain development using both zebrafish and mouse models. We found that cathepsin B expressing microglia were enriched in brain regions and time periods with high neuronal turnover including the zebrafish optic tectum and the murine somatosensory cortex. Zebrafish with myeloid-specific cathepsin B loss of function had dysmorphic microglia that acidified cargo at a slower rate, together with an accumulation of dead cells in the brain and impaired locomotor behavior. Consistent with this, mice with cathepsin B loss of function (*Ctsb*^*−/−*^) had impaired microglial phagocytosis of apoptotic cells. These mice displayed an accumulation of apoptotic cells in layer 5 of the somatosensory cortex at postnatal day 5, a region with pronounced neuronal turnover. Furthermore, cathepsin B deficiency in mice led to a decrease in cortical excitatory neuron density in the somatosensory cortex and tactile hypersensitivity. Taken together these data show that cathepsin B is necessary for effective microglial efferocytosis during brain development.

## Results

### Cathepsin B is a lysosomal protease that influences microglial morphology and density in the developing zebrafish optic tectum (OT)

**Fig. 1:**
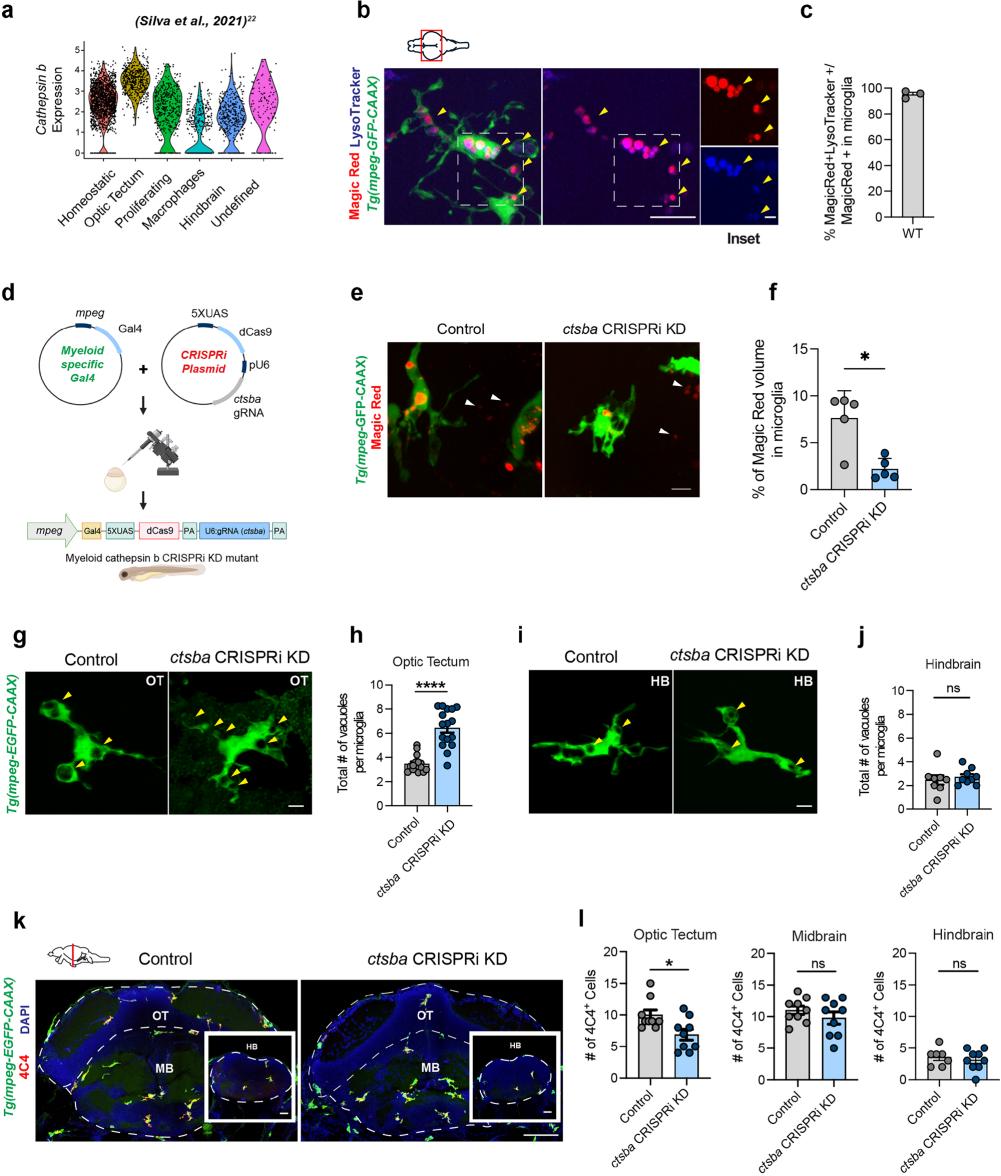
Cathepsin B is a lysosomal protease that influences microglial morphology and density in the developing zebrafish optic tectum (OT). **a** Expression of the zebrafish cathepsin B gene *(ctsba)* in microglial subsets identified in (Silva et al.)^22^ at 28 days post fertilization (dpf). **b** Representative image of an OT microglia (*Tg(mpeg1.1:GFP-CAAX)+*) exposed to cathepsin B substrate Magic Red-(RR)2 and acidification marker LysoTracker at 10 dpf. Yellow arrowheads indicate cathepsin B+ lysosomes co-labeled with Magic Red and LysoTracker. Scale = 10 µm. Inset scale = 5 µm. **c** Percent cathepsin B-Magic Red signal colocalized with LysoTracker within GFP+ microglia. *n* = 3 fish, averaging 2-3 microglia per fish. **d** Strategy for myeloid-specific CRISPRi knockdown (KD) of *ctsba*. See methods. Created in BioRender. Molofsky (2026) https://BioRender.com/544aq3i. **e** Representative images of cathepsin B-activity based on substrate Magic Red-(RR)2 in microglia from control and *ctsba* CRISPRi KD fish mutants. Scale = 10 µm. **f** Percent Magic Red within microglia from control and *ctsba* CRISPRi mutants. *n* = 5 fish, 2-3 microglia per fish. Two-tailed Welch’s *t*-test, *P = 0.010. **g** Representative images of control and *ctsba* CRISPRi KD fish in the OT at 10 dpf. Yellow arrowheads indicate vacuoles. Scale = 10 µm. **h** Vacuoles per microglia in OT control and *ctsba* CRISPRi KD fish. *n* = 16 fish, 2-3 microglia per fish. Two-tailed Mann–Whitney ****P = 0.0001. **i** Representative images of control and *ctsba* CRISPRi KD microglia in the HB. Yellow arrowheads = vacuoles. Scale = 10 µm. **j** Number of vacuoles per microglia in hindbrain (HB) control and *ctsba* CRISPRi KD fish. *n* = 8 fish, 2-3 microglia per fish. Two-tailed Welch’s *t*-test, ns, *P* = 0.56. **k** Representative images of microglia co-labeled with 4C4 (microglia marker) in the OT and HB from control and *ctsba* CRISRPi KD fish at 10 dpf. Scale = 20 µm. **l** Quantifications of 4C4+ microglia in the OT, MB, and HB at 10 dpf. *n* = 9 fish, 2-3 microglia per fish. Two-tailed Welch’s *t*-test, *P = 0.0174 (OT), ns (MB), and ns (HB). Values are mean $\pm \,$SEM. See also Supplementary Fig. S1. Illustration in b and k created using adobe illustrator.

We previously identified two molecularly distinct populations of microglia in the developing zebrafish. One subset was enriched in a synapse-rich hindbrain region and expressed genes associated with microglial synaptic remodeling, whereas a microglial subset in the optic tectum (OT), a region with high neuronal turnover, was enriched for multiple cathepsins^22^. The top differentially expressed gene enriched in OT microglia was the lysosomal protease cathepsin B (*ctsba*; Fig. 1a, Supplementary Fig. S1a)^22^. To define whether microglia had functional cathepsin B activity in vivo, we imaged live anesthetized fish after brain injection of the cathepsin B peptide substrate MR-(RR)2 (hereafter referred to as “Magic Red”), which becomes fluorescent after proteolytic cleavage by cathepsin B^12^. We also delivered LysoTracker dye to label acidified compartments (Fig. 1b)^12,23^. Microglia were identified by expression of a membrane localized GFP using the myeloid transgenic reporter *Tg(mpeg:GFP-CAAX)*. We observed cathepsin B activity (Magic Red + ) primary in microglia in the OT and found that cathepsin B activity was almost entirely observed within Lysotracker+ acidified compartments (Fig. 1c).

To study the function of cathepsin B during brain development, we generated a cell-type specific knockdown in zebrafish myeloid cells using CRISPR interference (CRISPRi; Fig. 1d). The GAL4-UAS system^24^ was used to target expression of GAL4 to microglia and myeloid cells using the *mpeg* promoter. The mpeg-GAL4 transcription activator protein binds to upstream activation sequence (UAS) to induce the expression of endonuclease-dead Cas9 protein (dCas9) and a guide RNA (gRNA) sequence targeting the *ctsba* gene under the ubiquitous U6 promoter. The fish were reared to homozygosity, and all experiments were conducted on F3 progeny. To validate knockdown of *ctsba*, we quantified Magic Red in controls (no transgene) and *ctsba* CRISPRi KD. We observed a 3.45-fold reduction in Magic Red volume within microglia of *ctsba* CRISPRi KD compared to controls (Fig. 1e, f). We further quantified expression of *ctsba* from flow isolated microglia by qPCR, which revealed a 37% reduction in *ctsba* transcript relative to housekeeping gene *ef1a* (Supplementary Fig. S1b, c). Given this relatively modest reduction in transcript, we compared our CRISPRi KD method to pharmacologic inhibition of cathepsin B using the cell-permeable inhibitor, CA-074 Me. The drug was delivered at a dose of 100 µm by swimming the fish for 3 days with daily replacements of the drug. We observed that either CRISPRi KD or pharmacologic inhibition led to a similar fold-decrease in MR fluorescence, but that the combination of these two did not have an additive effect (Supplementary Fig. S1d, e). We speculate that cathepsin B may be critical for microglial function, such that below a certain threshold of activity, a proportion of microglia may escape suppression or be replaced. Nevertheless, we observed substantial functional reduction of cathepsin B in microglia.

We next examined the impact of cathepsin B knockdown on microglial morphology at 10 days post fertilization (dpf) in the OT. We observed that *ctsba* deficient microglia in the OT had more vacuoles (defined as membrane enclosed compartments >2 µm in diameter; Fig. 1g, h) compared to controls. *ctsba* CRISPRi KD microglia had no difference in microglial volume (Supplementary Fig. S1f). Interestingly, vacuole number and microglia volume were unchanged in synapse-associated hindbrain microglia (Fig. 1i, j, Supplementary Fig. S1g), suggesting that cathepsin B could be more functionally relevant in the OT, where neuronal turnover is high.

We also quantified microglial numbers. We observed a ~25% reduction in the number of microglia in the OT but no differences in the midbrain and hindbrain as assessed by the microglia-specific marker 4C4 (Galectin 3)^25^ (Fig. 1k, l) at 10 dpf. By 20 dpf, microglia numbers in the OT were not different from controls (Supplementary Fig. S1h, i). This transient reduction in microglial numbers in *ctsba* CRISPRi KD fish could reflect impaired survival of microglia at a period when phagocytic demand is particularly high, although further experiments would be required to test this hypothesis. Together, our results suggest that cathepsin B is required for typical microglial morphology and density in the developing OT where neuronal turnover is high but is dispensable in synapse rich midbrain and hindbrain regions.

### Loss of microglial cathepsin B results in accumulation of dead cells and impaired locomotor behavior

**Fig. 2:**
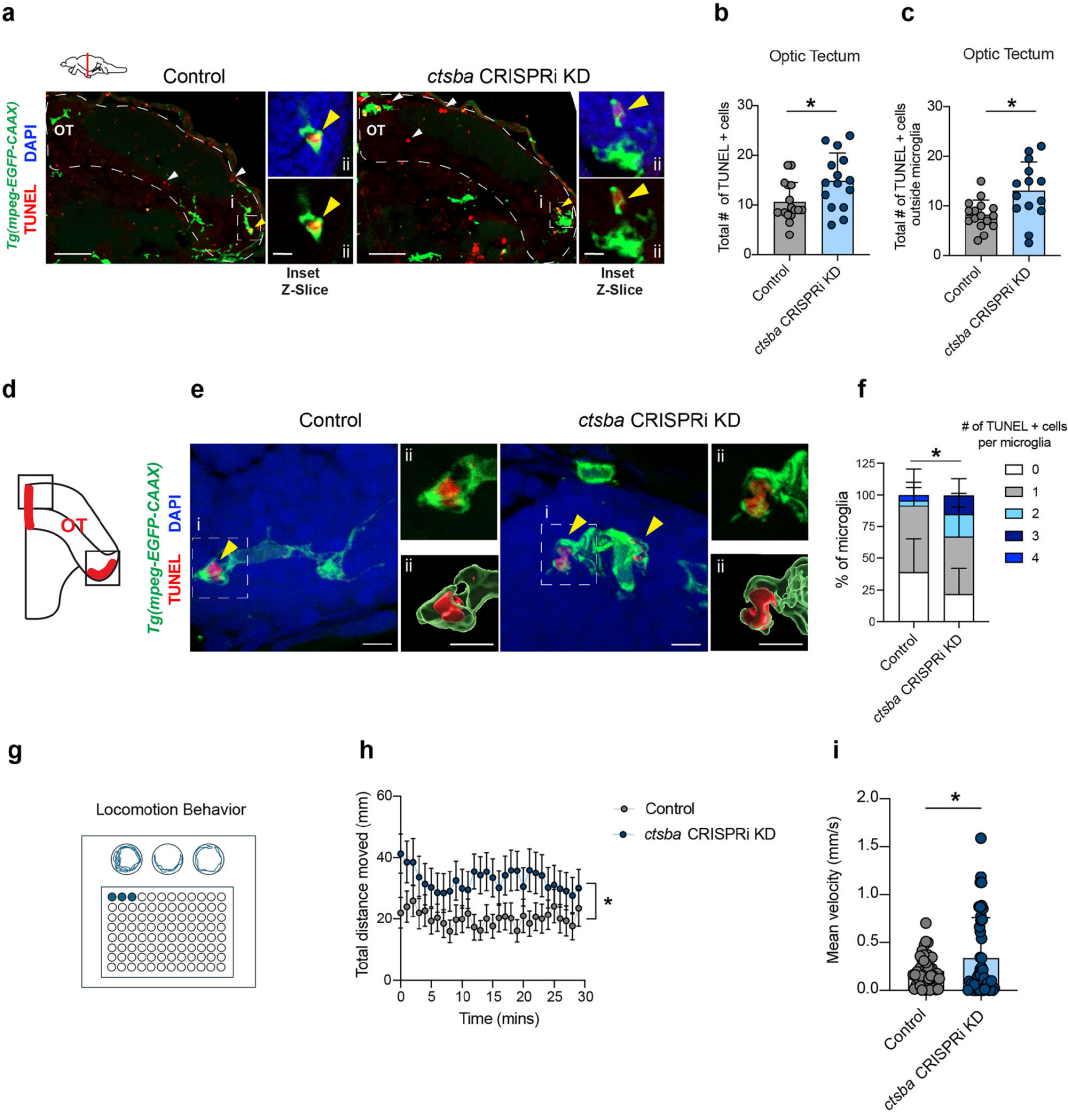
Loss of microglial Cathepsin B results in accumulation of dead cells and impaired locomotor behavior. **a** Representative images of microglia labeled with *Tg(mpeg:GFP-CAAX)* and apoptotic debris detected by TUNEL (terminal deoxynucleotidyl transferase-mediated deoxyuridine triphosphate nick end labeling) in the OT from controls and *ctsba* CRISPRi KD fish. White arrowheads indicate TUNEL+ cells outside of microglia. Yellow arrowheads in the inset indicate TUNEL+ cells in microglia. (i) Scale = 50 µm. (ii) Inset scale = 5 µm. **b** Quantification of total TUNEL positive cells in the optic tectum at 10 dpf. *n* = 15 fish, 2-3 microglia per fish. Two-tailed Welch’s *T* test, *P = 0.0228. **c** Quantification of percent TUNEL positive cells outside microglia from controls and *ctsba* CRISPRi KD fish in the OT. *n* = 15 fish, 2-3 microglia per fish. Two-tailed Welch’s *T* test, *P = 0.0126. **d** Schematic diagram of regions examined in (**e**, **f**). Red shading indicates regions of neuronal turnover in the OT. **e** Representative images of microglia containing TUNEL+ debris (yellow arrowheads from controls and *ctsba* CRISPRi KD fish. Insets show raw image (top) and 3D reconstruction (bottom). (i) Scale = 5 µm. (ii) Inset scale = 8 µm. **f** The number of TUNEL positive cells within microglia. Data represented as stacked bar graphs. *n* = 12 fish, 2-3 microglia per fish. Two-tailed Fisher’s exact test, *P = 0.0263. **g** Schematic diagram of 96 well plate used to swim larvae and record locomotion. **h** Locomotion behavior quantified as distance traveled (mm) from control and *ctsba* CRISPRi KD fish. *n* = 44 fish per group. 2-way RM ANOVA, *P = 0.0475, F (1,86) = 4.041. **i** Mean velocity in control and *ctsba* CRISPRi KD fish. Two-tailed Welch’s *T* test, *P* = 0.0421. *n* = 44 fish per group. Values were plotted as mean $\pm \,$SEM. Illustration in (**a**, **d**, **g**) created using adobe illustrator. See also Supplementary Fig. S2.

Microglia in the OT are critical for efferocytosis of neurons that die during the process of neurogenesis and neuronal turnover^26–28^. To examine if microglial cathepsin B was necessary for this process, we performed TUNEL staining (terminal deoxynucleotidyl transferase-mediated deoxyuridine triphosphate nick end labeling) to detect dead cells in control and microglial *ctsba* CRISPRi KD fish. We found that *ctsba* CRISPRi KD fish had an increase in the total number of TUNEL+ cells in the OT (Fig. 2a, b). We also observed an accumulation TUNEL+ cells outside (Fig. 2c) and within individual microglia in the OT (Fig. 2d–f). To complement this genetic approach, we used pharmacologic inhibition^29^ and quantified TUNEL+ cells in the OT at 10 dpf. The drug was delivered at a dose of 100 µm by swimming the fish for 3 days with daily replacements of the drug. We obtained concordant results to the *ctsba* CRISPRi KD fish using the CA-074 Me inhibitor (Supplementary Fig. S2a–c). These results suggest that loss of cathepsin B activity in microglia leads to reduced accumulation of dead neurons.

We also examined synapse numbers by staining for synaptic vesicle 2 (SV2) at 5, 10, and 20 dpf. We found that SV2 intensity was similar in control and *ctsba* CRISPRi KD fish at 5 and 10 dpf. However, SV2 intensity decreased in control fish at 20 dpf, but failed to do so in KD fish (Supplementary Fig. S2f, g). These changes in synapse density manifest after the period of robust cell death, resembling the temporal sequence of neuronal elimination followed by synapse pruning which is well documented in the mammalian brain. Together these results suggest that microglial cathepsin B is necessary for efficient clearance of dead cells and could also contribute to later synaptic remodeling in the developing brain. It is also possible that cathepsin B deficiency leads to an increase of cell death via a direct toxic effect of cathepsin B on neurons.

To assess whether microglial cathepsin B could impact brain function, we recorded spontaneous locomotion of zebrafish larvae at 10 dpf using a high throughput automated imaging platform (Fig. 2g)^30^. Following habituation to the recording platform, we recorded spontaneous movement for 30 min. We found that *ctsba* CRISPRi KD were hyperactive compared to controls as evidenced by an increase in total distance traveled and increased mean velocity (Fig. 2h, i). Thus, myeloid-specific depletion of cathepsin B leads to altered locomotor behavior, which could reflect several aspects of cathepsin B function, including its effects on cell death or synapse numbers, or secondary impairments of microglia’s typical homeostatic functions, such as regulation of neuronal excitability.

### Loss of cathepsin B impairs lysosomal acidification and digestion of neuronal corpses

**Fig. 3:**
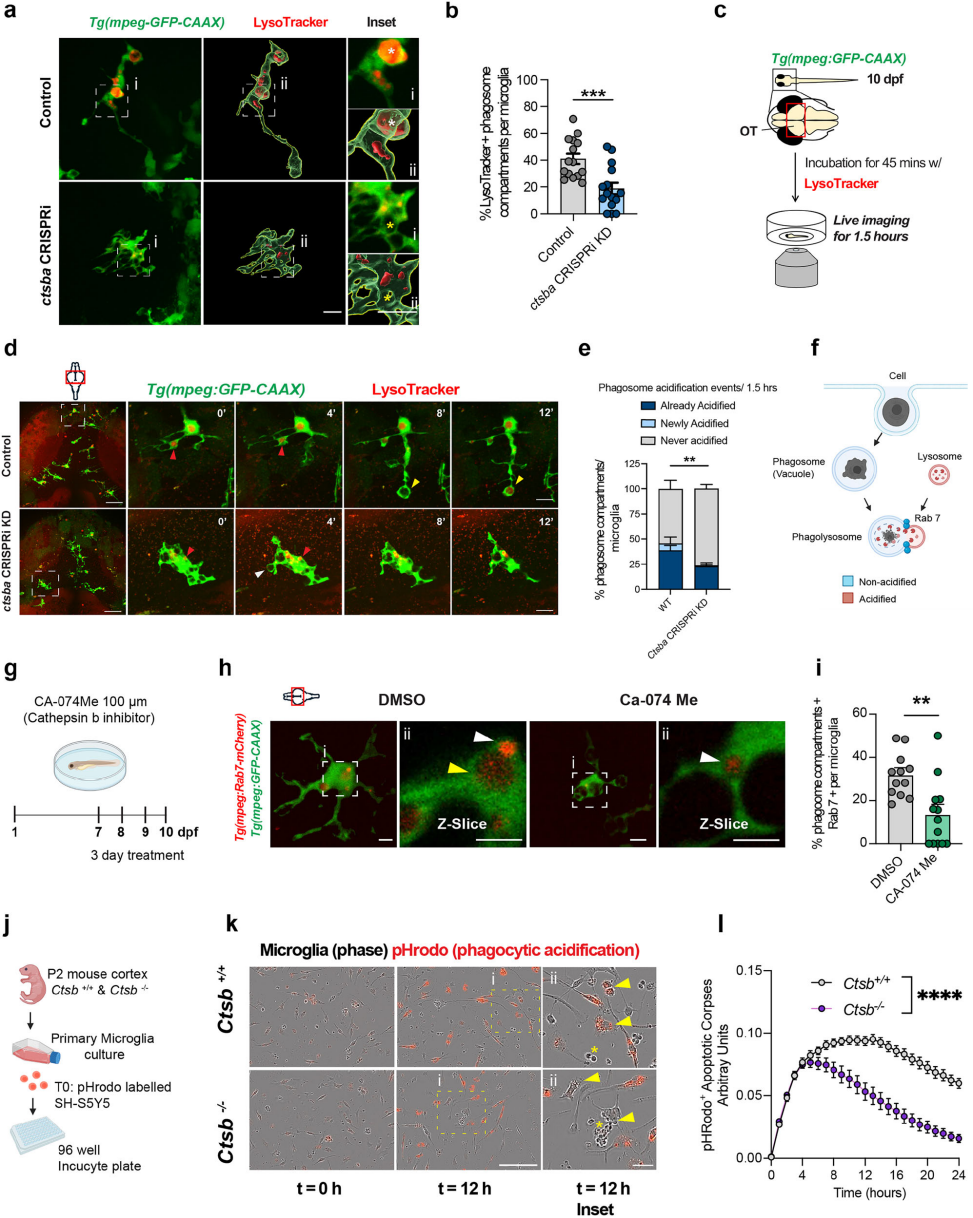
Loss of cathepsin B impairs lysosomal acidification and digestion of neuronal corpses. **a** Representative images of microglia Tg(*mpeg1.1-GFP-CAAX)* and LysoTracker signal in control and *ctsba* CRISPRi KD fish at 10 dpf. Scale = 10 µm. Inset: White asterisk, LysoTracker + phagosome and yellow asterisk, LysoTracker – phagosome. Scale = 7 µm. **b** Percent acidified phagosomes in control and *ctsba* CRISPRi KD fish. *n* = 16 fish, 2-3 microglia per fish. Two-tailed Welch’s *t*-test, ***P = 0.0004. **c** Schematic of zebrafish soaked in LysoTracker and live-imaging experiment. **d** Time series of microglia (green) and acidified compartments (Red, LysoTracker). Insets: microglia over time. Red arrowhead = already acidified. Yellow arrowhead = newly acidified, and white arrowhead = never acidified. Low power, scale = 20 µm. Inset, scale = 10 µm. **e** Acidification events during imaging session for control and *ctsba* CRISPRi KD fish. *n* = 5–6 fish, 8–10 microglia per fish. Two-tailed Fisher’s exact test, **P = 0.0065. **f** Schematic of stages of phagocytosis. reated in BioRender. Molofsky, A. (2026) https://BioRender.com/oewup89. **g** Schematic diagram of the pharmacological treatment with CA-074Me (cathepsin B inhibitor) vs. DMSO control. Created in BioRender. Molofsky, A. (2026) https://BioRender.com/pktvag5. **h** Representative images of microglia (green*)* and Rab 7 signal (red, *Tg(mpeg:Rab7-mcherry))* at 10 dpf. (i) Insets. (ii) z-slice, Rab7-mCherry protein. Yellow arrowheads indicate Rab7-mCherry + phagosome. Scale = 10 µm. **i** Percent of phagosomes that are Rab 7-mCherry+ within microglia from DMSO and CA-074Me. *n* = 10 fish, 2-3 microglia per fish. Two-tailed Welch’s *T*-test, **P = 0.0018. **j** Schematic of in vitro assay to quantify microglial phagocytosis. See methods. Created in BioRender. Molofsky, A. (2026) https://BioRender.com/mmxxjdq. **k** Representative images of primary microglial cultures at time = 0 and 12 from *Ctsb*^*+/+*^ and *Ctsb*^*−/−*^ groups. (i) corresponds to insets. (ii) yellow arrows show acidified SH-S5Y5 cells within microglia. Yellow asterisk shows undigested SH-S5Y5 cells. Scale = 10 µm. **l** Intensity curves from *Ctsb*^*+/+*^ and *Ctsb*^*−/−*^ primary microglial cultures fed with pHrodo ^+^ labeled apoptotic cells. 2-way RM ANOVA, ****P = 0.0001, F (1, 24) = 36.04. Values were plotted as mean $\pm \,$SEM. Illustration in (**c**, **d**, **h**) created using Adobe Illustrator. See also Supplementary Fig. S3, S4.

Efferocytosis, or engulfment of entire apoptotic cells, requires a robust and effective process of phagocytosis. Defects at any stage of this process could impair many features of phagocytosis, including engulfment, acidification, and digestion. Among these, acidification of phagocytic compartments is one of the most robust readouts due to the wide availability of pH sensitive dyes and molecules. We used LysoTracker dye to quantify acidification *ctsba* CRISRPi KD fish (Fig. 3a)^12,23^. We observed that despite the marked increase of phagocytic vacuoles in cathepsin B deficient microglia (Fig. 1h), there was a significant reduction in the percentage that were acidified (Fig. 3b) with a trend towards fewer total acidified compartments (Supplementary Fig. S3a). This did not appear to be driven by a reduction in lysosomes, as the number of putative lysosomes did not differ between control and *ctsba* CRISPRi KD fish (defined as LysoTracker+ and ≤2 µm in diameter; Supplementary Fig. S3b). We confirmed this result with the lysosomal marker LAMP1. We observed no difference in either number of LAMP1 positive lysosomes or total LAMP1 volume within microglia (Supplementary Fig. S3c–e). Together, our data suggests that loss of cathepsin B leads to a reduction in the number of acidified phagosomes with no change in lysosomal number and volume.

To more precisely examine the temporal dynamics of microglial efferocytosis and acidification, we used live imaging to quantify real-time acidification events within microglia (Fig. 3c, d). We classified microglial phagocytic vacuoles over the 90-min imaging period as ‘Already acidified’, ‘Newly acidified’, or ‘Never acidified’ based on whether they were or became LysoTracker+ during the imaging window (Supplementary Fig. S3f; Supplementary Movie S1, S2). We observed that *ctsba* CRISPRi KD microglia had fewer ‘Already acidified’ and ‘Newly acidified’ compartments, and more ‘Never acidified’ compartments (Fig. 3e). The morphology of these events also differed. Control microglia extended long processes that formed phagocytic compartments that subsequently acidified. In contrast, *ctsba* CRISPRi KD microglia had minimal process motility but could still form phagocytic cups. Interestingly, most acidified compartments were observed near the microglial soma in *ctsba* CRISPRi KD fish, which is consistent with microglia using alternative strategies of phagocytosis^31^. In separate experiments, we directly examined microglial motility in *ctsba* CRISPRI KD microglia compared to controls in the OT. We found that extension and retraction of microglial processes and total number of microglial processes were decreased in *ctsba* CRISPRi KD microglia (Supplementary Fig. S3g–i) correlating morphological defects (increased vacuoles; Fig. 1h) with altered motility dynamics.

An essential step in the phagocytic process is fusion of the phagosome containing phagocytosed material with an acidic lysosome filled with pH sensitive proteases such as cathepsins^8,10^. The site of this phagolysosome fusion is marked by Rab7 protein, a small GTPase that is associated with maturation of the phagosome (Fig. 3f)^32^. To test whether fusion of lysosomes to phagosomes was altered, we quantified Rab 7, which localizes to lysosomes and late endosomes using the transgenic reporter *Tg(mpeg:Rab7-mCherry)*^23^. We used the inhibitor CA-074 Me to globally suppress cathepsin B function (Fig. 3g). We observed that cathepsin B blockade significantly decreased both the percent of microglia containing Rab7-mCherry+ phagosomes and the total Rab7-mCherry volume in microglia (Fig. 3h, i, Supplementary Fig. S3j). To determine if the Rab7 protein is associated with the phagosome or lysosome, we quantified the number of putative phagosomes (≧5 µm) and lysosomes (<2 µm) that were Rab7 positive within microglia from both groups. Interestingly, CA-074 Me microglia had a significant reduction in the number of putative phagosomes that were Rab7 positive, but no difference in lysosomes (Supplementary Fig S3k–m). Taken together, our live imaging data in zebrafish show that cathepsin B deficient microglia have impaired motility, acidification, and efferocytosis. However, these data cannot definitively address whether any of these phenotypes are a primary or secondary effect of cathepsin B deficiency.

As our in vivo live imaging can capture microglia at various stages in their digestive lifecycle, we decided to further examine phagocytosis by performing live imaging of cultured primary microglia from *Ctsb* deficient mice and their littermate controls. Both groups were fed apoptotic corpses labeled with the pH-sensitive dye pHrodo at time = 0 and followed by 24 h live-imaging and quantification of pHrodo fluorescence (Fig. 3j)^14^. We observed that *Ctsb*^*−/−*^ microglia acidified phagocytic corpses markedly less that wild type littermate controls (Fig. 3k–l, Supplementary Fig. S4a). We examined the initial slope of the acidification phase and a plateau phase, which we interpreted as a balance of engulfment, acidification, and resolution, followed by a gradual decline of fluorescence as digestion proceeded and the availability of apoptotic corpses became exhausted (Supplementary Fig. S4b). We observed that the initial slope of acidification did not differ between WT and *Ctsb*^*−/−*^ microglia, suggesting that in the initial phases of digestion, acidification can proceed normally (Supplementary Fig. S4c). *Ctsb*^*−/−*^ microglia reached a peak acidification capacity lower than WT counterparts, followed by a rapid decline in acidified compartments, whereas WT microglia maintained a prolonged plateau phase (Supplementary Fig. S4d, e). Taken together, these two metrics suggest acidification is impaired during phagocytosis over time. These data are consistent with a model whereby impaired digestion of apoptotic corpses lead to secondary phenotypes in microglia as undigested material accumulates, including impaired motility, acidification, and phagolysosomal fusion in subsequent rounds of phagocytosis. While this model is consistent with the known function of cathepsin B as a lysosomal protease, they do not rule out the possibility that cathepsin B could have independent effects on motility and acidification.

### Cathepsin B is necessary for effective microglial efferocytosis during mouse brain development

**Fig. 4:**
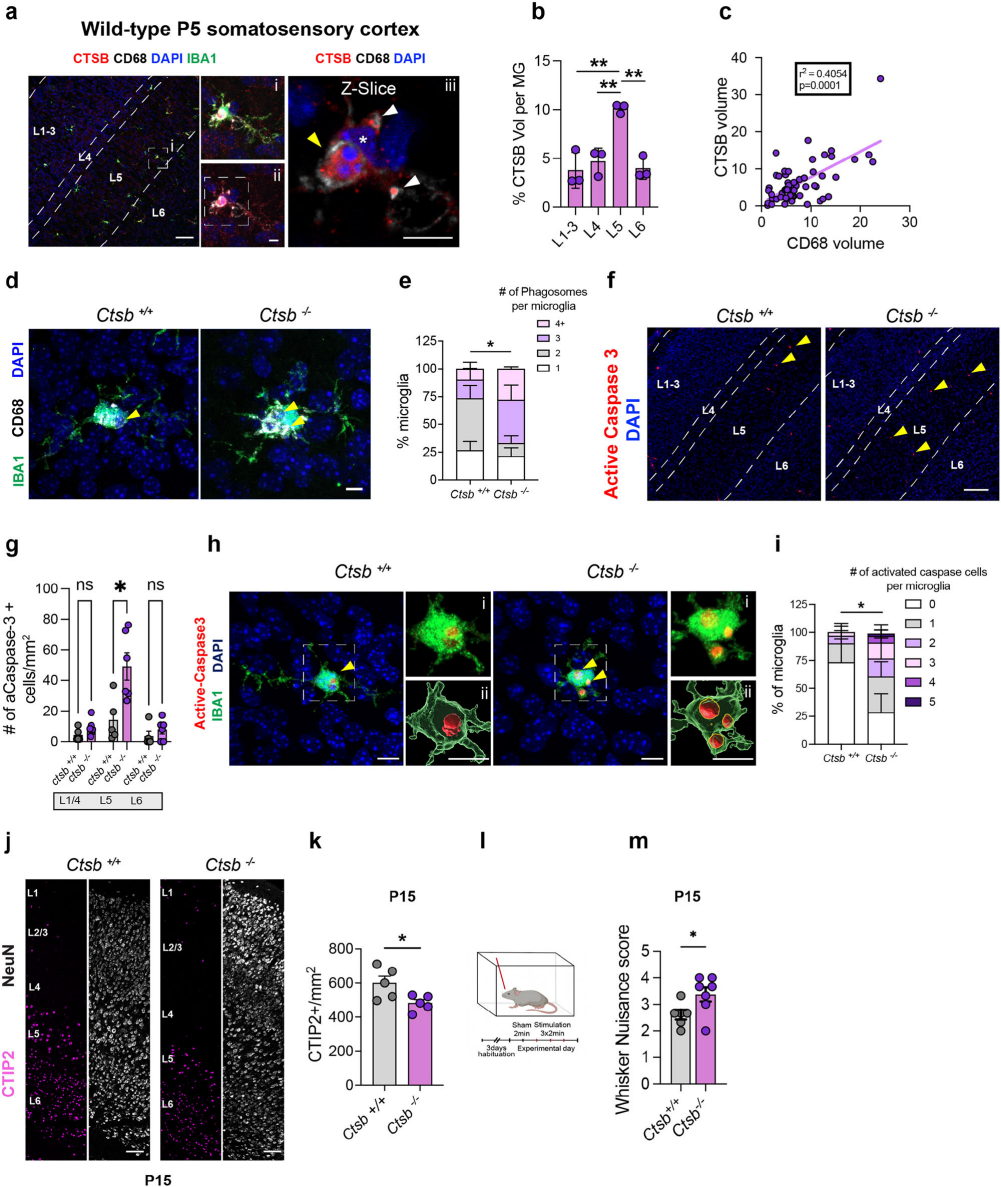
Cathepsin B is necessary for effective microglial efferocytosis during mouse brain development. **a** Representative images of somatosensory cortex microglia (IBA1+), cathepsin B (CTSB), CD68+ phagocytic compartments, and DAPI in postnatal day 5 (P5) mice. Scale = 100 µm. (i) all channels. (ii) without IBA1. (iii) z-slice. Yellow arrowhead: phagolysosome, white arrowheads: lysosomes, and asterisk: microglia nucleus. Scale = 5 µm. **b** Percentage of CTSB volume per microglia in L1-6 at P5. *n* = 3 mice. One way ANOVA with Tukey’s multiple comparisons, **P = 0.01. **c** Correlation between CD68 and CTSB volume per microglia at P5 (*n* = 3 mice, *n* = 59 microglia). Two-tailed r Spearman correlation coefficient = 0.40, *P* = 0.0001). **d** Representative images of CD68+ phagolysosomes co-stained with IBA1 from *Ctsb*^*+/+*^ and *Ctsb*^*−/−*^ mice at P5. **e** Quantification of the number of phagosomes per microglia. Phagosomes were defined as CD68+ vacuoles ≥3 µm in diameter. *n* = 4–5 mice, 2-3 microglia per mouse. Two-tailed Fisher’s exact test, *P = 0.0462. **f** Representative images of active caspase 3+ cells in the somatosensory cortex from *Ctsb*^*+/+*^ and *Ctsb*^*−/−*^ mice at P5. *n* = 5–6 mice/group. Scale = 50 µm. **g** Quantification of active-caspase 3+ cells within L1-L6 of the somatosensory cortex at P5. *n* = 5 mice/group. 2-way RM ANOVA with Sidak’s multiple comparisons, *P = 0.0349. **h** Representative images of active-caspase 3+ cells within microglia in L5 from *Ctsb*^*+/+*^ and *Ctsb*^*−/−*^ mice. Yellow arrowheads: active-caspase 3 inside microglia. (i) raw image. (ii) 3D reconstruction. Scale = 20 µm. **i** Number of active-caspase 3+ cells within microglia from *Ctsb*^*+/+*^ and *Ctsb*^*−/−*^ mice. *n* = 4–5 mice/group, 2-3 microglia per mouse. Two-tailed Fisher’s exact, **P = 0.0066. **j** Representative images of CTIP2 and NeuN+ neurons in *Ctsb*^*+/+*^ and *Ctsb*^*−/−*^ mice at P15. Scale = 50 µm. **k** Total CTIP2^+^ neuron density in *Ctsb*^*+/+*^ and *Ctsb*^*−/−*^ mice at P15. *n* = 5 mice per group. Two-tailed Welch’s *t*-test, *P = 0.0436. **l** Schematic of whisker nuisance assay. (see methods). Created in BioRender. Molofsky, A. (2026) https://BioRender.com/5ijrzgo. **m** Whisker nuisance score in *Ctsb*^*+/+*^ and *Ctsb*^*−/−*^ mice (*n* = 5–8 mice/group) at P15. Two-tailed Welch’s *t*-test, *P = 0.0375. Values were plotted as mean $\pm \,$SEM. See also Supplementary Figs. S5, S6.

Cathepsin B is a core microglia gene expressed across species and highly enriched in microglia relative to other brain cells in both human and mouse (Supplementary Fig. S5a, b)^21,33^. We therefore examined whether the developmental function of cathepsin B was conserved in mice. We focused our studies on the early postnatal rodent somatosensory cortex (postnatal day 5, P5) during a peak of high neuronal turnover and where we previously observed microglial-mediated neuronal elimination selectively within cortical layer 5 (L5)^14^. Consistent with our findings in fish, we found that cathepsin B protein was primarily detected in microglia, using an antibody validated in *Ctsb*^*−/−*^ tissue (Supplementary Fig. S5c). Consistent with a role in cellular clearance, cathepsin B was strongly enriched in L5 relative to other cortical layers, (Fig. 4a, b) and returned to baseline levels in the somatosensory cortex at P15 when neuronal apoptosis is low (Supplementary Fig. S5d, e). Within L5 microglia, we found that cathepsin B staining colocalized with CD68, a membrane protein found on lysosomes, late endosomes, and phagolysosomes (Fig. 4c)^34^. To next determine the function of cathepsin B in murine brain development, we examined mice globally deficient for cathepsin B (*Ctsb*^*−/−*^). We found more CD68+ compartments per microglia in *Ctsb*^*−/−*^ compared to *Ctsb*^*+/+*^ littermate controls (Fig. 4d, e), as well as an increase in CD68 volume (Supplementary Fig. S4f) which persisted at P15 (Supplementary Fig. S5 g, h). Microglia number was unchanged at P5 (Supplementary Fig. S5i, j) and P15 (Supplementary Fig. S5 k, l). These data indicate that cathepsin B is predominantly expressed in microglia and is necessary for effective microglial phagocytosis in murine cortex.

Due to the buildup of dead cells observed in *ctsba* CRISPRi KD fish, we quantified apoptosis in the murine cortex using an antibody for active caspase-3^35^. We found that *Ctsb*^*−/−*^ mutants had an increase in total apoptotic cells which was specific to L5 (Fig. 4f, g). We also observed an accumulation of apoptotic material within microglia (Fig. 4h, i), as well as an increase in TUNEL+ material (Supplementary Fig. S5m, n). To determine if the accumulation of apoptotic material persisted to P15, we quantified active caspase-3 and detected no significant increase in any layer within the cortex (Supplementary Fig. S4o, p). We did not observe a difference in the reactive astrocyte marker GFAP in *Ctsb*^*−/−*^ cortex compared to littermate controls, arguing against more generalized toxicity associated with glial reactivity (Supplementary Fig. S6a, b), although further experiments would be required to more definitively assess this. Taken together, our results indicate that cathepsin B regulates microglial efferocytosis of neurons in the developing mouse brain during critical waves of neuronal apoptosis.

Finally, we examined the impact of cathepsin B on mouse cortical development and function. Since our effects were predominantly in L5, we quantified the excitatory neuron subtypes in this region using the markers CTIP2 and SATB2^14^. During peak apoptosis (P5) we did not observe a difference in the number of CTIP2+, SATB2+, double positive neurons, or total neurons (NeuN+) (Supplementary Fig. S6c–g). However, by P15 the density of CTIP2+ neurons in L5/6 were significantly reduced (Fig. 4j, k). There was no change in the overall number of total neurons (NeuN+) or upper layer excitatory neurons (SATB2+) in *Ctsb*^*−/−*^ mice (Supplementary Fig. S6h, i) at P15, suggesting this effect was specific to layer 5 excitatory neurons, where apoptotic cell death and phagocytic microglia were observed at P5. To test if cathepsin B impacted somatosensory behavior, we used a whisker nuisance assay at P15 to measure aversive behavioral responses to tactile stimulation using a standardized rating scale (Table 1)^36,37^, as we previously optimized in juvenile mice^14^. We observed a significant increase in tactile hypersensitivity in *Ctsb*^*−/−*^ mice (Fig. 4l, m). Taken together, these data reveal that cathepsin B is important for somatosensory circuit maturation and function during mouse cortical development, although we cannot definitively establish whether in mice this is due to a cell-autonomous function of cathepsin B in microglia.

**Table 1:** Whisker nuisance behavioral scoring system

	Score		
Behaviors	0	1	2
Response to stick	Interested or ignore	Avoiding, anxious	Attacks, bites
Evasiveness	No evasive behavior	Runs away <50%	Runs away, moves to protect whiskers
Grooming	Normal grooming	No grooming	Irritated scratching, rubbing, pulling
Freezing	Walks around, exhibits curiosity	Frozen <50% time	Frozen, defensive, fearful >50% time
Breathing	Normal range		Hyperventilating, gasping

## Discussion

**Fig. 5:**
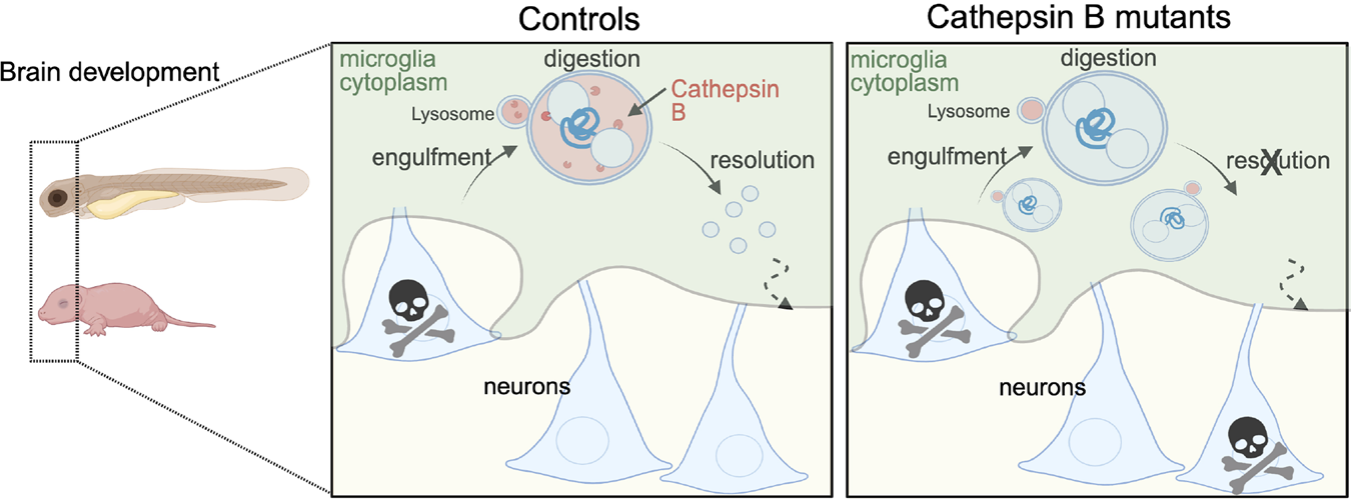
Summary diagram illustrating the role of cathepsin B in effective microglial efferocytosis during vertebrate brain development. Schematic of zebrafish larvae and postnatal mice brain from control and cathepsin B mutants. Control microglia engulf dead neurons, digesting the material upon lysosomal-phagosome fusion mediated by lysosomal protease cathepsin B. Finally, this process is resolved to maintain a healthy brain circuit and behavior. Cathepsin B mutants engulf dead neurons but fail to digest and acidify material in the phagosome resulting in an unrefined circuit and hyperactive behavior. Created in BioRender. Molofsky, A. (2026) https://BioRender.com/axfmpdz.

Microglial efferocytosis is vital for brain development and regeneration^7^. Our study identifies microglial cathepsin B as a critical contributor to the process of microglial efferocytosis that is required for typical neural circuit development (Fig. 5).

A major finding from our study is that cathepsin B deficiency leads to phagocytic defects that manifest in a buildup of non-acidified vacuoles within microglia. This raises the question of how a protease that is largely active at acidic pH prevents a buildup of non-acidified compartments. The most parsimonious explanation is that since cathepsin B is a lysosomal protease, it directly impacts digestion (although we cannot rule out an unexpected role). This impaired proteolysis may contribute to many of the secondary phenotypes that we observed, including reduced numbers of acidified compartments and impaired motility. Impairment at any stage of the digestion process can reduce the efficiency of phagocytosis, eventually contributing to impaired resolution of the phagolysosome, which is vital to regenerating resources needed for subsequent rounds of phagocytosis^38^. This places cathepsin B in a similar category to other mechanisms that promote orderly and efficient phagocytosis, including v-ATPases that promote acidification^12^, mechanisms of gastrosome maturation^13^, and cation channels that are required for phagosome resolution^11,39^.

Another key finding from our study is that loss of microglial cathepsin B led to a buildup of dead cells in developing brain that was closely correlated with the accumulation of dysfunctional microglia. One interpretation of this is that impaired efferocytosis leads to the accumulation of dead neurons and subsequent behavioral phenotypes. However, we cannot rule out that microglial cathepsin B deficiency could increase cell death and affect behavior through other mechanisms. For example, under conditions of proteotoxic stress, some cells may eject toxic contents via exophers, thereby causing damage to bystander cells,^40^. Our mouse studies clearly identified these cells as apoptotic based on active caspase-3 staining (Fig. 4f–i). Many other pathways involved in lysosomal function lead to similar accumulations of neuronal debris^23,41,42^. For example, microglia lacking the lysosomal GTPase RagA have an increase in phagosomes and an accumulation of neuronal debris^23,41^. Mutations of the *mcoln1* in zebrafish and drosophila lead to increased neuronal death^43,44^ and visual and motor deficits also seen in patients with mucolipidosis type IV^45^. These findings suggest that cathepsin B’s role in microglia phagocytosis is necessary for healthy brain development, consistent with our findings of behavioral deficits in both zebrafish and mouse models of cathepsin B deficiency.

While cathepsin B is highly specific to microglia within the brain, our knockdown of cathepsin B in zebrafish also targets other myeloid cells, thus we cannot rule out that global phenotypes, such as behavior, are partly due to peripheral macrophage effects. Similarly, our mouse mutant targets cathepsin B in all cells including neurons. The limited temporal resolution of our imaging may also have missed more rapid events, such as fine process motility and transitions between stages of phagocytosis.

Finally, it is notable that the requirement for cathepsin B in microglial efferocytosis was most pronounced in regions with high neuronal turnover, including the zebrafish optic tectum (relative to midbrain and hindbrain) and the mouse somatosensory cortex in layer 5 (relative to other cortical layers). These data suggest that while cathepsin B is not the only mechanism promoting microglial phagocytosis, it is particularly relevant in settings of high phagocytic demand, such as whole cell efferocytosis. This is consistent with other studies. Mice lacking cathepsins B and L show an increase in apoptosis and a decrease of neurons in some brain regions^46^ and *Ctsb*^*−/−*^ mouse embryonic fibroblasts have cholesterol accumulation and lysosomal dysfunction similar to what is observed in Niemann-Pick type C disease (NPC)^47^. While cathepsin B itself is not associated with any known human lysosomal storage disorder, Cathepsin B activity is increased in NPC disease^48,49^, suggesting that it could play some compensatory role. This implies that unlike in neurodegeneration, where cathepsin B has been proposed to be pathogenic,^50^ it is possible that promoting cathepsin B function during development could be protective in neurodevelopmental diseases.

## METHODS

### Zebrafish

Animal protocol: AN205699 was approved by the University of California, San Francisco (UCSF) and in accordance with the guidelines established by the Institutional Animal Care and Use Committee and Laboratory Animal Resource Center. Wild-type, AB strain zebrafish (*Danio rerio*; ZIRC, University of Oregon, Eugene, OR) were propagated, maintained, and housed in recirculating habitats at 28.5 °C and on a 14/10-h light/dark cycle. Embryos were collected after natural spawns, incubated at 28.5 °C and staged by hours post fertilization (hpf). Larvae used were 10 days post fertilization (dpf), a time in development before sex determination. Ages were matched within experiments. The transgenic reporter lines, *Tg(mpeg1.1:EGFP-CAAX)*^51^ was used to mononuclear phagocytes and *Tg(mpeg1.1:Rab7-mCherry)*^23^ to label Rab7 expression in myeloid cells.

### Mice

All mouse strains were maintained in the University of California San Francisco specific pathogen–free animal facility, and protocol number AN203917 was approved by and in accordance with the guidelines established by the Institutional Animal Care and Use Committee and Laboratory Animal Resource Center. Mice were housed in a 12-h light/dark cycle (7am-7pm) at 68–79 °F and 30–70% humidity. Male and female mice were group-housed, when possible, with up to 5 mice per cage. Littermate controls were used for all experiments when feasible. The following mouse strain used is referenced in the text as *Ctsb*^*−/−*^: [B6;129-Ctsb^tm1Jde^*/J]* (Jax# 030971) mice^52^ were backcrossed six times to *C57BL/6J* and were obtained from Dr. Jason G. Cyster, University of California, San Francisco, CA. In all experiments, both male and female mice were used with ages specified in the legends.

### Magic red injections

10 dpf larvae fish were injected with 2 nL of Magic Red at a dilution of (1:260) directly into the brain and returned to system water immediately following injections. Fish were imaged 24 h post injection using the Nikon CSU-W1 spinning disk/high speed widefield microscope. We took images from the optic tectum collecting 40–60 µm z-stacks (step size: 0.5 µm). The images were processed by FIJI software.

### Generation of *ctsba* cell-type specific zebrafish mutant

The tol2 based mpeg:Gal4-VP plasmid was a gift from Dr. Sarah Kucenas and co-injected with the UAS:CRISPRi-U6:*ctsba* plasmid and transposase mRNA for integration. To generate the UAS:CRISPRi-U6:*ctsba* plasmid, we modified the backbone (psELN04 (pTol1-uas:dcas9-PA,pu6:gRNA) from Dr. Cody Smith at the University of Notre Dame (cloning: https://invivobiosystems.com/) to add the following gRNA *ctsba* sequence: ACCATCTCATGGGACAAGGGAGG. The CRISPRi gRNA was selected using the CRISPR-ERA design tool. Casper embryos were injected at the 1 cell stage with 12.5 nl/μl of plasmid and 25 ng/μl of transposase mRNA. F1 mutant hybrids were in crossed to establish F2 homozygous generation. Screening was carried out by FACS and qPCR described below. The primers used for screening were: Fwd: 5’CTTCCGTGATGTGGACTACAGC-3’ Rev: 5’AGACGGGTATCCACCATTAC-3’.

### Fluorescence activated cell sorting (FACS)

For cell-type specific CRISPRi validation, 10 dpf *Tg(mpeg1.1:EGFP)* zebrafish brain were dissected (10 zebrafish were pooled per sample). Briefly, the brains were mechanically dissociated in isolation medium (1× HBSS, 0.6% glucose, 15 mM HEPES, 1 mM EDTA pH 8.0) using a glass tissue homogenizer (VWR). Subsequently, the cell suspension was filtered through a 70 µm filter (Falcon) and pelleted at 300 × *g*, 4 °C for 10 minutes. The pellet was resuspended in 22% Percoll (GE Healthcare) and centrifuged at 900 × *g*, 4 °C for 20 min (acceleration set to 4 and deceleration set to 1). Afterwards, the myelin free pellet was resuspended in isolation medium that did not contain phenol red. Prior to sorting on a BD FACS Aria III, cell suspension was incubated with DAPI (Sigma). *mpeg* + cells were collected for qPCR by gating on FSC/SSC scatter, live cells by DAPI, and all *mpeg*:GFP cells.

### Quantitative PCR (qPCR)

To extract RNA from cells isolated by FACS, freshly sorted cells were pelleted at 500 × *g* for 10 min at 4° and then resuspended in RLT Plus buffer (Qiagen 1053393). Cells were vortexed and frozen for at least one day at −80° before being thawed on ice and processed for RNA using a RNeasy Plus Mini Kit (Qiagen). Purified mRNA was converted to cDNA with the High Capacity cDNA Reverse Transcription kit (Life Technologies) and amplified using either the Fast SYBR Green Master Mix (Thermo Fisher 43-856-12) and a 7900HT Fast Real-Time PCR System (Applied Biosystems).

### Immunohistochemistry

For mouse brain tissue collection, mice were transcardially perfused with 5–10 mL of sterile 1X PBS followed by 5–10 mL of 4% paraformaldehyde. Tissues were fixed overnight at 4 °C in 0.1 M phosphate buffered 4% paraformaldehyde, cryoprotected with 20% sucrose, and embedded in optimal cutting temperature (OCT) medium (Sakura Finetek USA, Torrance, CA). Immunohistochemistry (IHC) was performed using 16 to 25-μm-thick sections were collected and mounted onto glass slides or 40 µm thick floating sections were collected into 0.1 M PBS. Fish brain sections were washed in phosphate buffer saline with 0.5% Triton-x (PBST) and incubated with 20% heat-inactivated normal goat serum in PBST for 2 h (NSS; Sigma-Aldrich, Corp.). Mouse brain sections were blocked in staining buffer (5% normal goat serum, 0.4% Triton-X, 1X PBS) for 1 h. Primary antibodies in staining buffer were applied overnight at 4 °C. Sections were then washed with PBST three times and incubated in secondary antibodies for 2 h at room temperature (Silva et al.). Sections were washed with PBST three times and mounted with DAPI Fluoromount-G (SouthernBiotech). Stained and mounted sections were imaged on an LSM700 confocal microscope (Zeiss) using ×20 (numerical aperture 0.8) and ×63 (numerical aperture 1.4) objectives. Images were analyzed with ImageJ (v.2.14.0).

For CTIP2 and SATB2 immunostaining (antibody information below), sodium citrate antigen retrieval was performed. Briefly, sections were immersed in fresh sodium citrate buffer (10 mM sodium citrate, pH 6.0) at 98 °C for 10 min and cooled at room temperature for 20 min. IHC was performed as described above.

Antibodies were: chicken anti-GFP (Aves Labs, cat# GFP-1020, 1:1000), Living Colors DsRed (Clontech, cat# 632496, 1:1000), mouse anti-SV2 (DSHB, AB2315387, 1:500), mouse anti-4C4 (gift from Hitchcock lab, 1:200), guinea pig anti-Iba1 (Synaptic Systems, cat# 234-004, 1:1000), chicken anti-NeuN (Millipore, cat# ABN91, 1:500), rat anti-CD68 (Bio-Rad, cat. no. MCA1957GA, 1:500), rat anti-CTIP2 (Biolegend, cat# 650601, 1:500), rabbit anti-SATB2 (Abcam, cat#AB92446, 1:500), rabbit anti-active Caspase-3 (BD Pharmingen, cat#559565, 1:500), rabbit anti-cathepsin B (Abcam cat# ab214428, 1:500). All secondary antibodies were used at 1:500 dilution and from ThermoFisherScientific: goat anti-chicken Alexa Fluor (AF) 488 (cat#A-11039), goat anti-mouse AF 488 (cat#A-11001), goat anti-mouse AF 555 (cat#A-11003), goat anti-mouse AF 647 (cat#A-21236), goat anti-rabbit AF 555 (cat# A-21428), goat anti-rat AF 555 (cat#A-21434).

### TUNEL staining

TUNEL staining was performed using In Situ Cell Death Detection Kit, TMR red (Sigma-Aldrich, CAT# 12156792910). Glass mounted sections were postfixed at 4 °C for 20 min prior to blocking. Following secondary antibody incubation, slides were permeabilized with PBS containing 1% sodium citrate/ 1% Triton-X-100 at 4 °C, rinsed with PBS, and then were incubated with TUNEL reaction cocktail per kit instructions for 1 h at 37 °C.

### Confocal microscopy on fixed tissues

Images were acquired with a Zeiss LSM 800 laser scanning confocal microscope with 405, 477, 561, 650 nm laser lines. For mouse sections, 8-bit images were acquired with 5× scan speed at 1024 × 1024 resolution, 0.8 µm Z step size, and 2X line averaging. Laser power and gain were consistent within experiments.

### Microglia engulfment assay

Images were acquired with an LSM 800 Confocal Microscope (Zeiss) using the same parameters as described above. Imaris software (Bitplane) was used to generate 3D surface rendering of microglia, which were then masked for TUNEL or active caspase 3 channels within those microglia. Masked channels were then 3D rendered to obtain volume data. TUNEL or active caspase 3 engulfment was calculated per cell as the volume of the respective signal divided by the volume of the microglia.

### Microglia CD68 volume

Z-stacks were collected on an LSM 880 confocal microscope with AiryScan (Zeiss) on Superresolution mode and a 63× objective (NA 1.4). Laser power and gain were consistent across each image. AiryScan processing was performed in Zen software (Zeiss) at a setting of 6 (“optimal” setting). Images were analyzed using Imaris software (Bitplane) by creating a 3D surface rendering of individual microglia, thresholded to ensure microglia processes were accurately reconstructed, and maintained consistent thereafter. Microglia rendering was used to mask and render the CD68 channel within each microglia. CD68 volume per microglia was then calculated as the total volume of masked CD68 volume within the masked GFP volume.

### Neuronal subtype counts

Images of the somatosensory cortex from P15 mice were acquired using 20× magnification (NA 1.0) and 5×1 tiling to cover all cortical layers in a single optical section. NeuN staining was used to determine the center Z stack plane of the tissue section. All images were acquired in the same anatomical region of the somatosensory cortex, using the dorsal hippocampus as a reference point. Using FIJI image analysis software, ROIs were drawn for each cortical layer using DAPI staining. NeuN, CTIP2, and SATB2 analysis was automated followed by manual correction. Images were first segmented to binary images using Moments thresholding for CTIP2 and Huang thresholding for SATB2. Prior to quantification, the water-shedding function was used to segment touching cells. Particle analysis was used to quantify positive neurons using a size restriction (size 20 μm-infinity). Neuronal counts were divided by the cortical layer area in mm^2^ to generate density per layer. 1–3 sections were imaged and quantified per mouse.

### Zebrafish live imaging

For live imaging, *Tg(mpeg:EGFP-CAAX)* zebrafish larvae incubated with LysoTracker (10 mM) for 45 min then were anesthetized with 0.2 mg/ml of tricaine in embryo medium and mounted in 1.2% low-melting agarose gel on a glass bottom 35-mm dish (MatTek) and covered with embryo water containing 0.2 mg/ml tricaine. Time-lapse image was performed on a Nikon CSU-W1 spinning disk/high speed widefield microscope. We took time-lapse images from the optic tectum collecting 40–60 µm z-stacks (step size: 0.5 µm) at 4 min intervals for 1 h and 30 min. The images were processed by FIJI software. For analysis, we grouped the acidification events into three categories: 1) Phagosomes that were ‘Already’ acidified indicated by LysoTracker positivity 2) Phagosomes that became ‘Newly’ acidified.

### Locomotion behavior

Behavior studies on 10 dpf larvae were conducted in a 96-well plate using the automated locomotion detection device Danio Vision system and EthoVision XT 11.5 software (DanioVision, Noldus Information Technology). Larvae were placed into the DanioVision chamber to habituate for 30 min before recording locomotion for 30 min. At least 40 larvae per group (control and *ctsba* CRISPRi mutants) were used for a total of 3 independent experimental trials.

### Primary microglia culture and engulfment assay

Mixed glia cultures were generated from P2 pups from *Ctsb*^*+/+*^ or *Ctsb*^*−/−*^ and grown in T75 flasks with DMEM (Gibco 11965126) supplemented with heat inactivated 10% FBS (Gibco 10437028) and 1% Pen/Strep (Gibco 15140122) at 37 °C 5% CO_2_. Media was changed the next day and cultures were grown for another 10–12 days. Microglia were detached by hitting the flasks 10× against the bench and plated in a 96-well plate at a density of 20,000 cells/well in 100 µl final volume (4–6 replicate wells per condition). Cells were stained with Trypan blue (Invitrogen T10282) and counted using a Countess 3 Automated Cell Counter (Invitrogen). The next day, microglia were treated with 10,000 pHRodo^+^ apoptotic corpses (ratio 1:2) were added to the microglia 10–15 min before the first image acquisition (T = 0 h). Images were acquired using an Incucyte S3 Live Cell Analysis Instrument (Sartorius) at 20×, 4 images per well, every hour for 24 h total in 555 nm red channel (apoptotic corpses within lysosomes) and phase contrast (microglia). Images were then thresholded using the Incucyte Software for Live Cell analysis and the integrated intensity of the red channel (apoptotic corpses within lysosomes) normalized to microglia surface area (determined with phase contrast) was used for analysis. This value was multiplied by 10^3^ and is plotted as “pHrodo intensity (arbitrary units)” in Fig. 3L and Supplementary Fig. S3D.

### Generation of apoptotic cells

The human neuroblastoma cell line, SH-S5Y5, was maintained in DMEM (Gibco 11965126) culture medium supplemented with 10% FBS and 1% Pen/Strep. To generate apoptotic corpses, cells were grown to 80% confluence and treated with 20 µM Navitoclax (Bcl-2 family protein inhibitor,^36^) for 24 h. Supernatant containing the dead cells was collected and centrifuged at 1000 × *g*/5 min/RT. Cells were resuspended in PBS at concentration of 10^6^/mL. 1 μl of 1 mg/ml pHrodo-SE (stock solution in DMSO, Thermo Fisher P36600) was added per 50 ml of cell suspension. Cells were incubated 30 min at RT before washing twice in PBS before resuspending them in DMEM supplemented with 10% FBS and 1% Pen/Strep. Apoptotic cells were used immediately for microglia engulfment assays.

### Behavioral assay – Whisker nuisance test

The whisker nuisance test paradigm was adapted from ref. ^27^. Briefly, mixed gender P15 littermate controls and CTSB KO mice were used for a minimum of 3 experiments. Each mouse was habituated to an individual cage on 3 consecutive days from P12 to P14. Using the wooden end of a long q-tip, we then performed a sham stimulation, presenting the q-tip in front of the head of the mouse without touching it for a duration of 2 min followed by 3 consecutive 2 min trials gently stroking the whiskers on the right side of the face continuously, separated by 1 min intervals. All trials were recorded with a camera for later analysis. Using the video recording, five categories of behavioral responses to the whisker stimulation were scored as detailed in the Table 1 below. These parameters reflect increased avoidance, fear and aggression towards the probe. Interpretation: Normal behavioral responses to stimulation were assigned a zero value, whereas meaningful abnormal behavioral responses were assigned a value of 2. The maximum whisker nuisance score is 10. High scores (6–10) indicate abnormal responses to the stimulation, in which the mouse freezes, becomes agitated or is aggressive. Low scores (0–3) indicate normal responses, in which the mouse is either soothed, curious or indifferent to the stimulation.

### Quantification and statistical analysis

Graphpad Prism 8.3.0 was used for all histological quantification analyses. Statistical tests are described in text and figure legends.

### Reporting summary

Further information on research design is available in the Nature Portfolio Reporting Summary linked to this article.

## Supplementary information

Supplementary Information

Description of Additional Supplementary Files

Movie S1

Movie S2

Reporting Summary

## Source data

Source Data

## Data Availability

Supplement contains additional data. All data needed to evaluate the conclusions in the paper are present in the paper or the Supplementary Materials. All additional information will be made available upon request to authors. Source data are provided with this paper.
