# Supplementary material for: Microglial cathepsin B is necessary for neuronal efferocytosis in zebrafish and mice during brain development
Source: Nat Commun. 2026 Mar 13;17:3881. doi: 10.1038/s41467-026-70350-1 (PMC13125305; doi:10.1038/s41467-026-70350-1)
Supplement: Supplementary file 1 — Supplementary Information [file 41467_2026_70350_MOESM1_ESM.pdf]

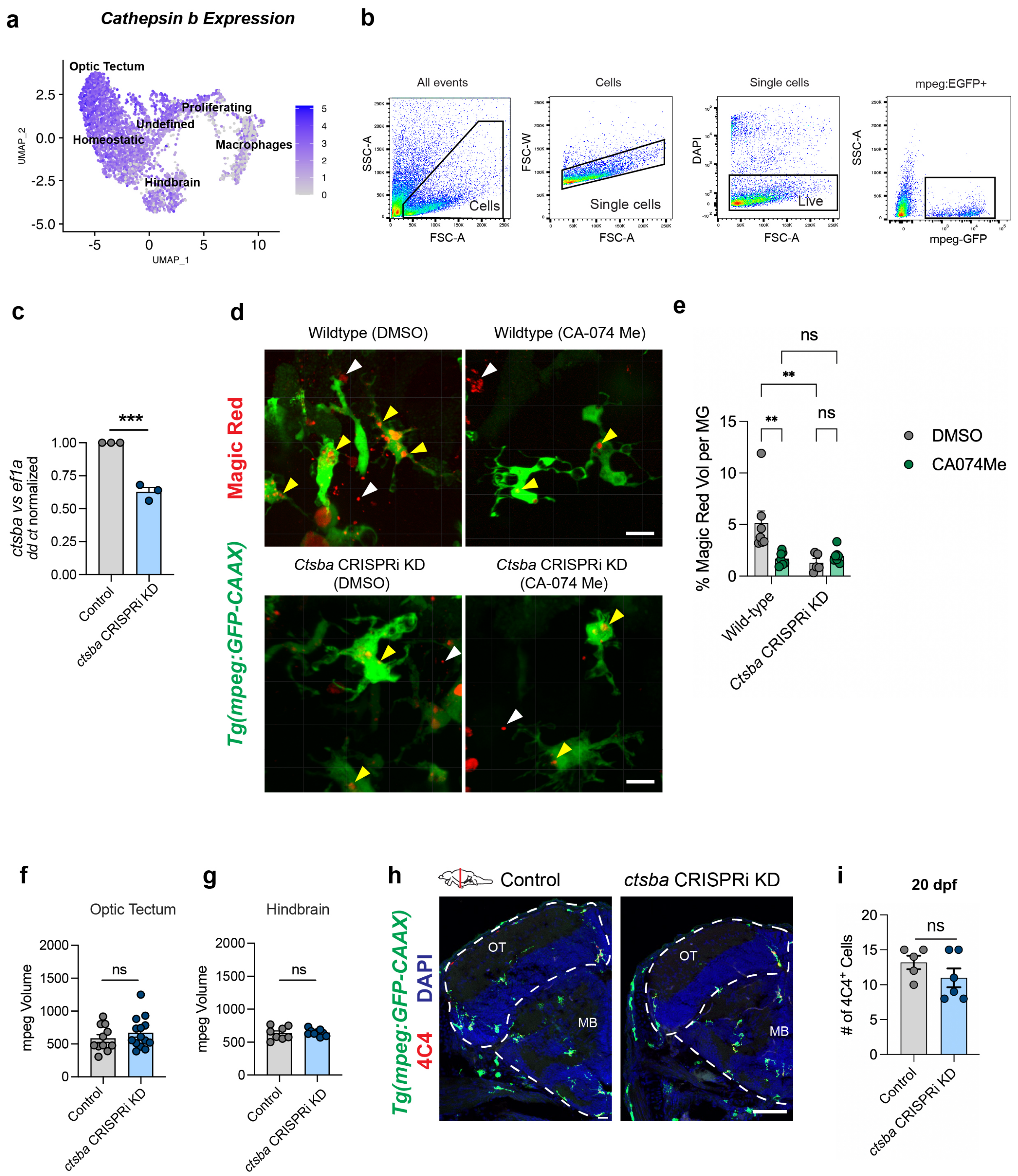

Figure S1: Validation of *ctsba* CRISPRi KD fish, related to Figure 1.

a Feature plot cathepsin B gene (*ctsba*) expression in UMAP space from 28 dpf zebrafish microglia from (*Silva et al, 2021)*[22].

b Flow cytometry gating strategy to isolate *mpeg1.1*-EGFP+ myeloid cells for qPCR from control and *ctsba* CRISPRi KD fish using whole larvae at 10 dpf.

c qPCR for *ctsba* expression of flow sorted *mpeg*+ cells from control and *ctsba* CRISPRi KD whole larvae at 10 dpf. Expression levels are normalized to housekeeping gene *ef1a*.

(Dots represent independent replicates, each pooled from 10-15 fish). Welch's t-test, *** P = 0.0005.
d Representative images of microglia exposed to cathepsin B substrate Magic Red (MR) in wild-type controls and *ctsba* CRISPRi KD fish with either DMSO or CA-074Me. White arrowheads: MR outside of microglia and yellow arrowheads: MR inside of microglia. Scale = 10 µm.
e Quantification of the % Magic Red within microglia from wild-type and *ctsba* CRISPRi KD with either DMSO or CA-074 Me. 2way ANOVA, ** P = 0.00313 (WT: DMSO and CA-074 Me), ** P = 0.0011 (WT: DMSO and *ctsba* CRISPRi KD). n = 5-7 fish/group, 3-5 microglia per fish.
f Quantification of OT mpeg volume from control and *ctsba* CRISPRi KD microglia. n= 12 fish/group, 2-3 microglia per fish. ns, P=0.0574.
g Quantification of HB mpeg volume from control and *ctsba* CRISPRi KD microglia. n= 9 fish/group, 2-3 microglia per fish. ns, P=0.8546.
h Representative images of microglia co-labeled with *Tg(mpeg1.1:GFP-CAAX)* and 4C4 (microglia marker) in the OT from control and *ctsba* CRISPRi KD at 20 dpf. Scale = 100 µm.
i Quantifications of 4C4+ microglia in the optic tectum at 20 dpf. n = 5-6 fish/group. Welch's t-test, ns.
Values were plotted as mean $\pm$ SEM. Illustration in h created using Adobe Illustrator.

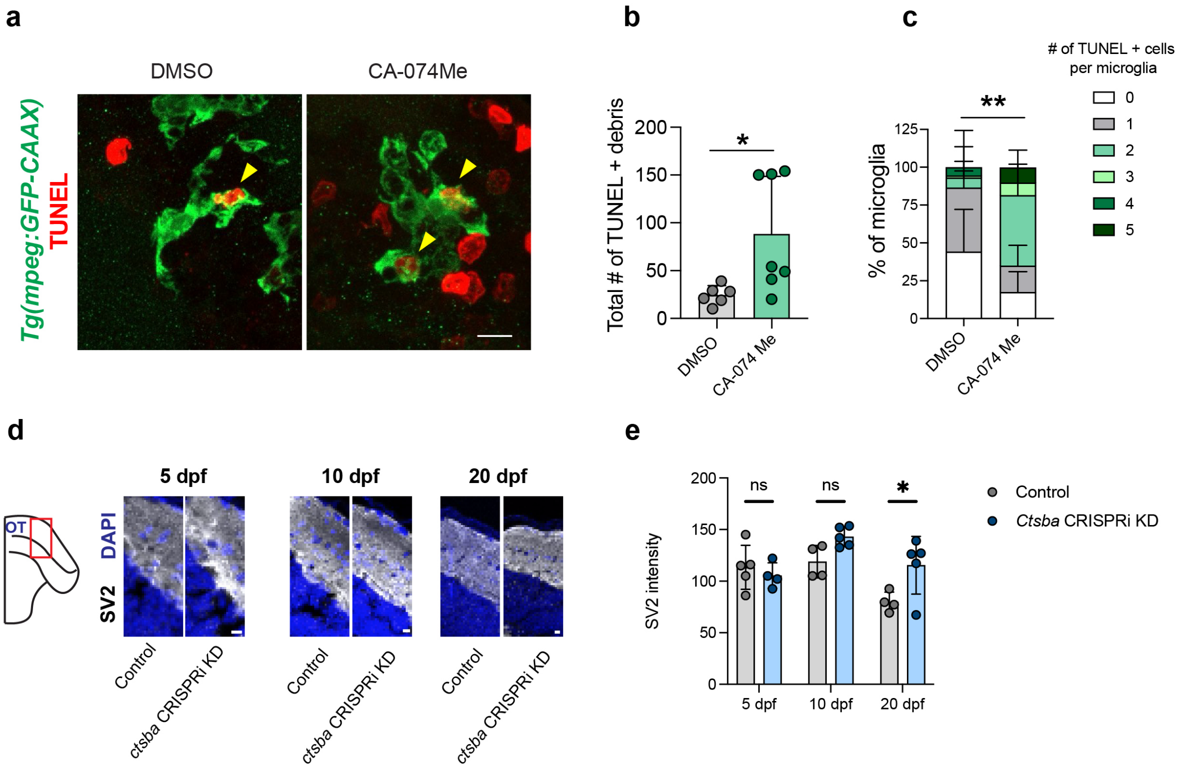

Figure S2: Impact of cathepsin B pharmacological inhibition on cell death and microglial efferocytosis, related to Figure 2.

a Representative images of dead cells detected by TUNEL (terminal deoxynucleotidyl transferase-mediated deoxyuridine triphosphate nick end labeling) and microglia *Tg(mpeg:EGFP-CAAX*) from DMSO and CA-074Me treated groups. Yellow arrowheads indicate TUNEL+ cells within microglia. Scale bar =10 μm.

b Quantification of the total number of TUNEL+ cells in OT from DMSO and the cathepsin B inhibitor CA-074Me treated groups at 10 dpf. n = 6 fish, 2-3 microglia per fish. Welch's t-test, * P = 0.0301.

c Quantification of the number of TUNEL+ cells within microglia from DMSO and CA-074Me treated groups in the optic tectum at 10 dpf. n = 6 fish, 2-3 microglia per fish. Fisher's exact test, ** P = 0.0052.

d Representative images of synaptic marker (SV2) in the optic tectum from control and Ctsba CRISRPi KD fish at 5, 10, and 20 dpf. Scale = 5 μm.

e Quantification of the total SV2 intensity in the optic tectum of control and *Ctsba* CRISRPi KD at 5, 10, and 20 dpf. 2way ANOVA, Sidak's multiple comparisons *P = 0.0225. n = 5 fish per group.

Values were plotted as mean ± SEM. Illustration in d created using Adobe Illustrator.

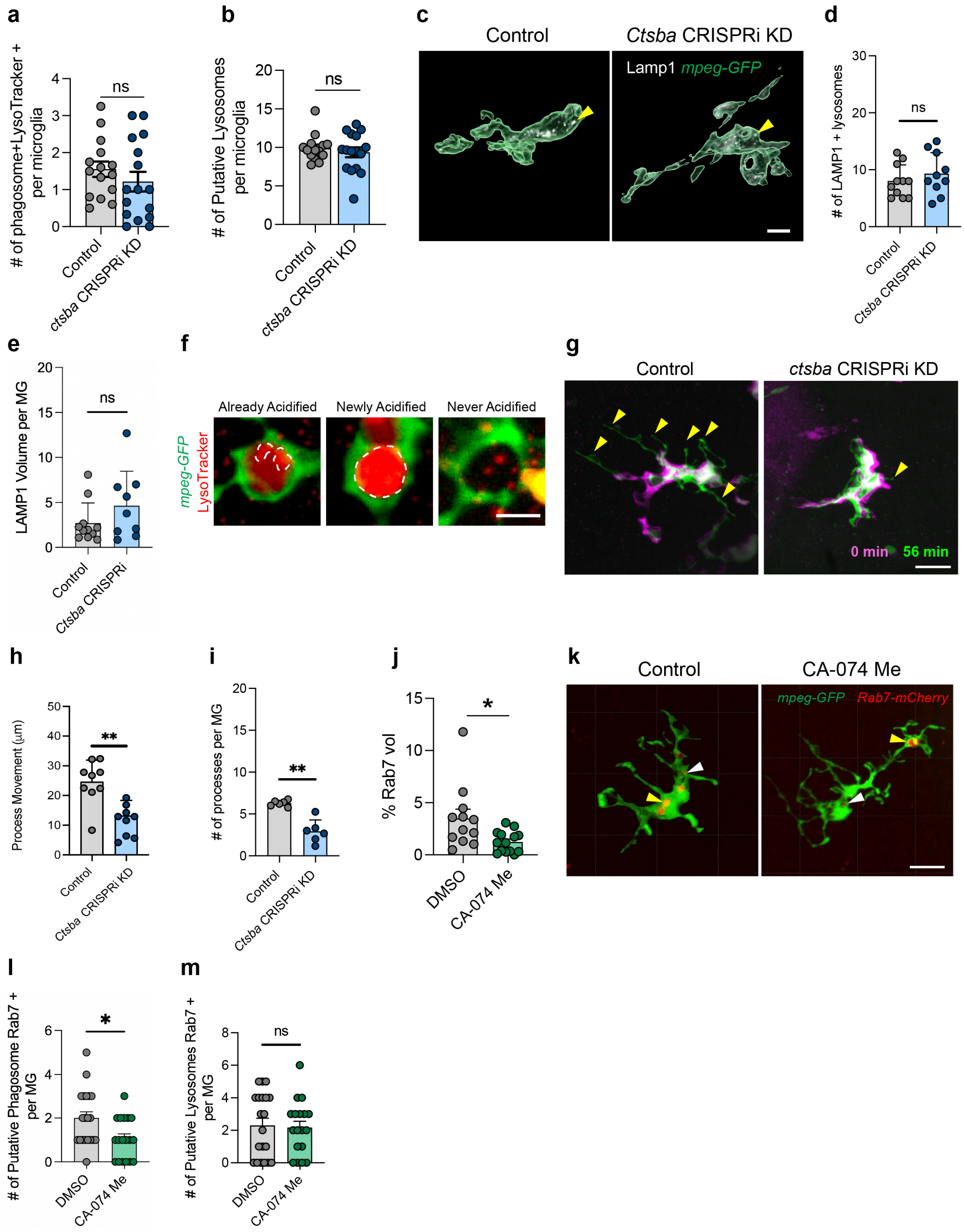

Figure S3: Characterization of microglial phagocytosis in *ctsba* CRISPRi KD fish, related to Figure 3.

a Number of acidified phagosomes (size>3µm) within microglia from control and *ctsba* CRISPRi KD fish. Welch's t-test, ns. n=16 fish, 2-3 microglia per fish.

b Number of putative lysosomes in control and *ctsba* CRISPRi KD fish. Size exclusion ≤ 2µm. Welch's t test, ns. n=16 fish, 2-3 microglia per fish.

c Representative images of lysosomal marker, LAMP1 in microglia from control and *ctsba* CRISRPi KD fish. Scale bar=10µm. Yellow arrowheads= lysosomes.

d The total number of lysosomes (LAMP1+) within microglia from control and *ctsba* CRISRPi KD fish. n= 4, 2-3 microglia per fish. Welch's t-test, ns.

e Total LAMP1 volume within microglia from control and *ctsba* CRISRPi KD fish. n=4, 2-3 microglia per fish. Welch's t-test, ns.

f Representative images of "already acidified", "newly acidified", and "never acidified" microglia;GFP and LysoTracker. Scale=5µm. Dashed line= condensed or brighter LysoTracker signal.

g Representative images of microglia from control and *ctsba* CRISRPi KD fish at timepoints 0 (Magenta) and 56 min (Green) frame. Scale=10µm. Yellow arrowheads indicate new processes at 56 min.

h Quantification of the total process movement (µm) in control and Ctsba CRISRPi KD fish over the course of an hour. n= 4, 2-3 microglia per fish. Welch's t-test, ** P =0.001.

i Quantification of total number of processes per microglia from control and *ctsba* CRISPRi KD. n= 4, 2-3 microglia per fish for both groups. Welch's t-test, ** P=0.001.

j Total volume of Rab7-mCherry expression within *Tg(mpeg1:GFP-CAAX*) positive cells from control and *ctsba* CRISPRi KD fish. n=12. Welch's t-test, *P = 0.0291. n=10 fish, 2-3 microglia.

k Representative images of microglia; green and *Rab7-mCherry (late endolysosome reporter)* in microglia from DMSO and CA-074Me fish. White arrowheads: Lysosomes and Yellow arrowheads: Phagosomes. Scale=10µm.

l The total number of putative phagosomes that are Rab7+ within microglia from DMSO and CA-074Me fish. n=4, 2-3 microglia. Welch's t-test, ns.

m The total number of putative lysosomes that are Rab7+ within microglia from DMSO and CA-074Me fish. n=6-7, 2-3 microglia. Welch's t-test, ns.

Values were plotted as mean ±SEM.

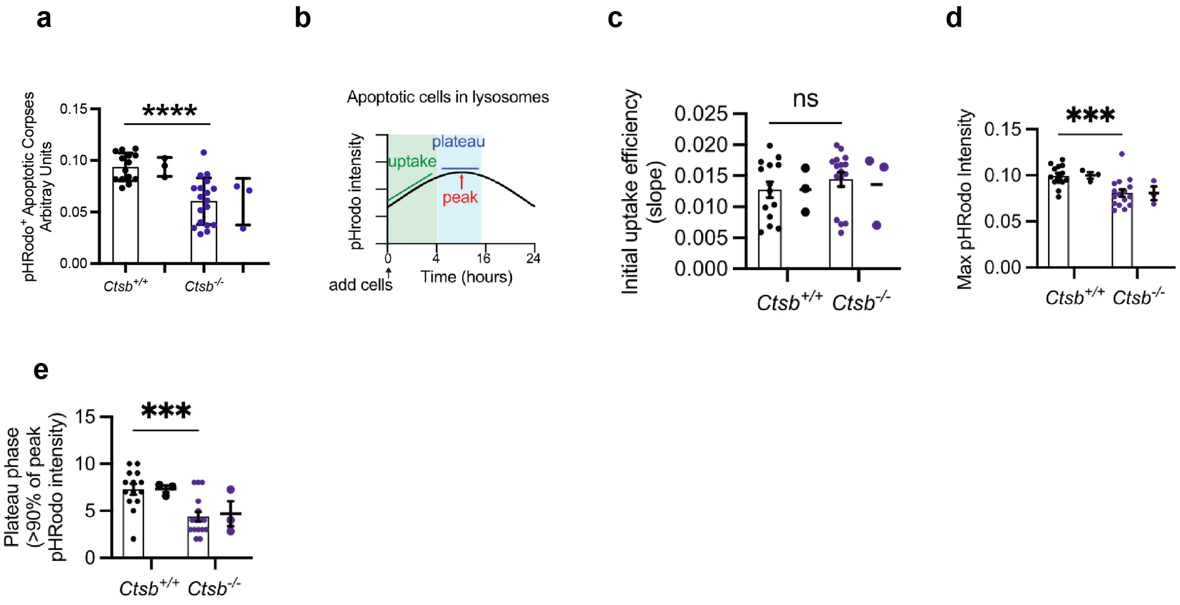

Figure S4: Characterization of microglial phagocytosis *in vitro* cell culture, related to Figure 3.

a Quantification of pHRodo$^+$ signal in primary cultured microglia from *Ctsb* $^{+/+}$ and *Ctsb* $^{-/-}$ mice at 12 hours following the seeding of pHrodo-labeled apoptotic SH-S5Y5 cells. Bars indicate technical replicates (n = 14 vs. 16 wells) from three independent experiments. Welch's t-test. ****P = 0.0001.

b Schematic of typical phagocytosis curve. A slope of linear regression of the pre-peak slope during the linear phase was used to histamine the update efficiency (green). Plateau phase (blue) is defined as >90% of peak signal intensity. Red arrow shows the peak of the curve.

c The slope of the curve between 0-4 hours after adding neuronal corpses (green in A). Bars indicate technical replicates (n = 14 vs. 16 wells) from three independent experiments (large dots show mean per experiment).

d Maximum pHrodo intensity at the peak of engulfment (red in A). Bars indicate technical replicates (n = 14 vs. 16 wells) from three independent experiments (large dots show mean per experiment).

e Duration of the plateau phase (blue in A) defined as the timepoints with at least 90% of peak pHrodo intensity (in hours). Bars indicate technical replicates (n = 14 vs. 16 wells) from three independent experiments (large dots show mean per experiment).

Values were plotted as mean ± SEM. Illustration in b created using Adobe Illustrator.

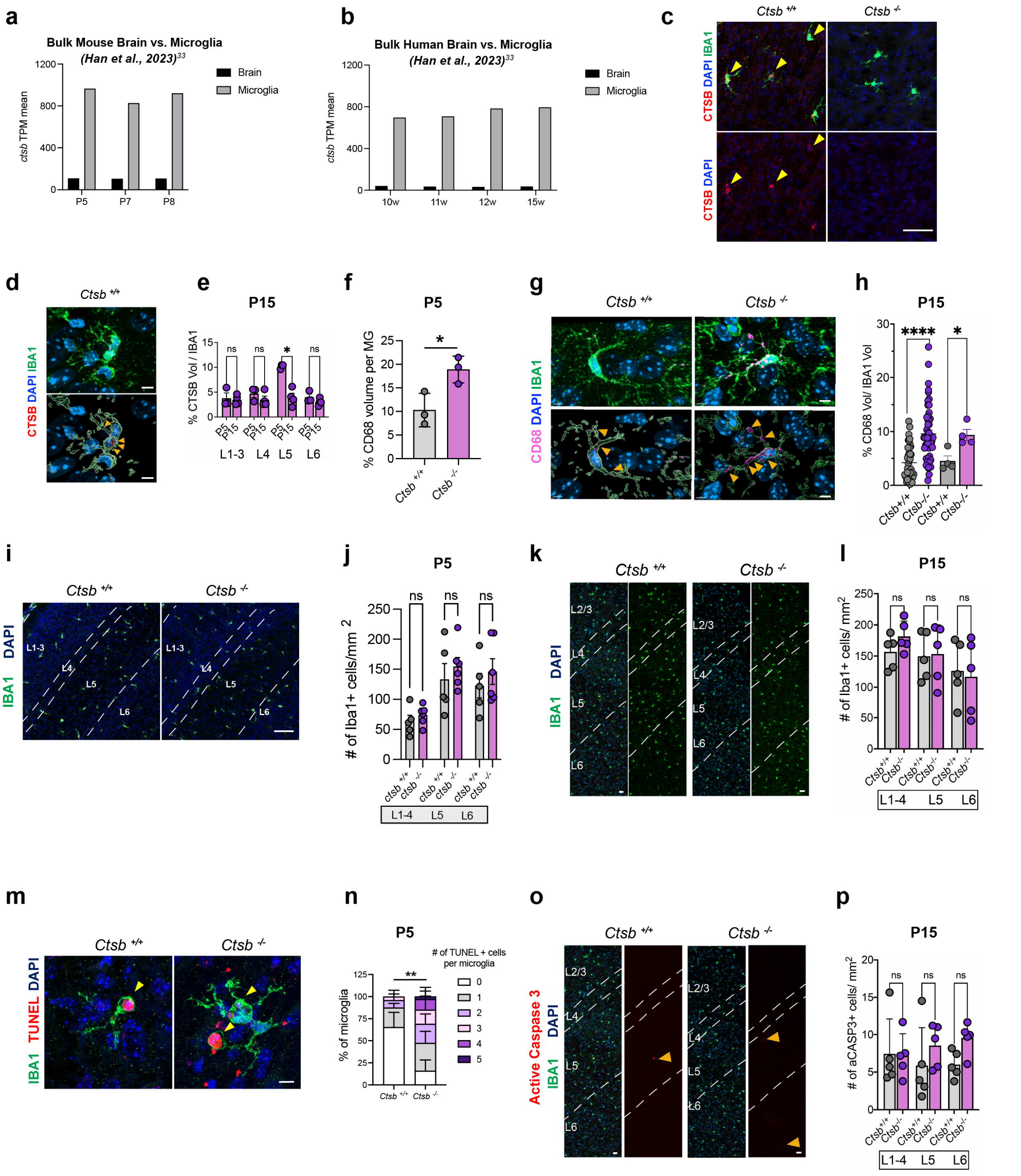

Figure S5: Additional characterization of *Ctsb*-deficient mice, related to Figure 4.
a *CTSB* bulk sequencing of mouse brain vs. isolated mouse microglia (TPM:transcripts per million) from *(Han et al., 2023)*[33].
b *CTSB* bulk sequencing of human fetal brain and microglia (w:gestational weeks) from (*Han et al., 2023)*[33].
c CTSB in somatosensory cortex from *Ctsb*$^{+/+}$ and *Ctsb*$^{-/-}$ mice at P5. Scale=25μm.

d Representative raw image and 3D rendered of CTSB (below) inside microglia (IBA1+, green) in L5 of the wildtype somatosensory cortex. Scale=4μm. Orange arrows= CTSB puncta.
e CTSB volume within IBA1 at P5 and P15. P5 data from Figure 4B. 2way ANOVA, *P=0.0129
f CD68 volume per microglia in the somatosensory cortex from *Ctsb*$^{+/+}$ and *Ctsb*$^{-/-}$ mice at P5. (n= 3 mice/group). Welch's t-test, *P=0.0327.
g Representative raw images (top) and 3D rendered (bottom) of microglia from *Ctsb*$^{+/+}$ and *Ctsb*$^{-/-}$ L5 of the P15. CD68 (magenta) inside microglia (green). Scale=5μm. Orange arrows= CD68 puncta.
h CD68 volume within microglia in L5 at P15 by cell (left) and animal (right). By cell, Welch's t test ****P=0.0001. By animal, unpaired t test *P=0.0129
i Representative images of IBA1^{+} staining (green) in the somatosensory cortex from *Ctsb*$^{+/+}$ and *Ctsb*$^{-/-}$ mice at P5. n=5-6 mice/group. Scale=50μm
j IBA1^{+} cells in layers L1-L5 within the somatosensory cortex from *Ctsb*$^{+/+}$ and *Ctsb*$^{-/-}$ mice at P5. n= 5 mice/group. 2-way RM ANOVA with Sidak's multiple comparisons, ns.
k Representative images of IBA1^{+} staining (green) in the somatosensory cortex at P15. Left, *Ctsb*$^{+/+}$ and right, *Ctsb*$^{-/-}$. Scale=20μm.
l Density of IBA1+ cells for each cortical layer at P15. 2way ANOVA.
m Representative images of TUNEL+ cells within microglia from *Ctsb*$^{+/+}$ and *Ctsb*$^{-/-}$ mice. Yellow arrowheads= TUNEL+ microglia. Scale= 5μm.
n Quantification of TUNEL positive cells within microglia from *Ctsb*$^{+/+}$ and *Ctsb*$^{-/-}$ mice at P5. Data calculated from means of 4-3 mice/group, 2-3 microglia per mouse. Fisher's exact test, **P=0.0066.
o Representative images of aCasp3+ cells (red) at P15 somatosensory cortex. Left, *Ctsb*$^{+/+}$ and right, *Ctsb*$^{-/-}$. Scale=20μm.
p Density of aCasp3+ cells for each cortical layer at P15. 2way ANOVA.
Values were plotted as mean$\pm$SEM.

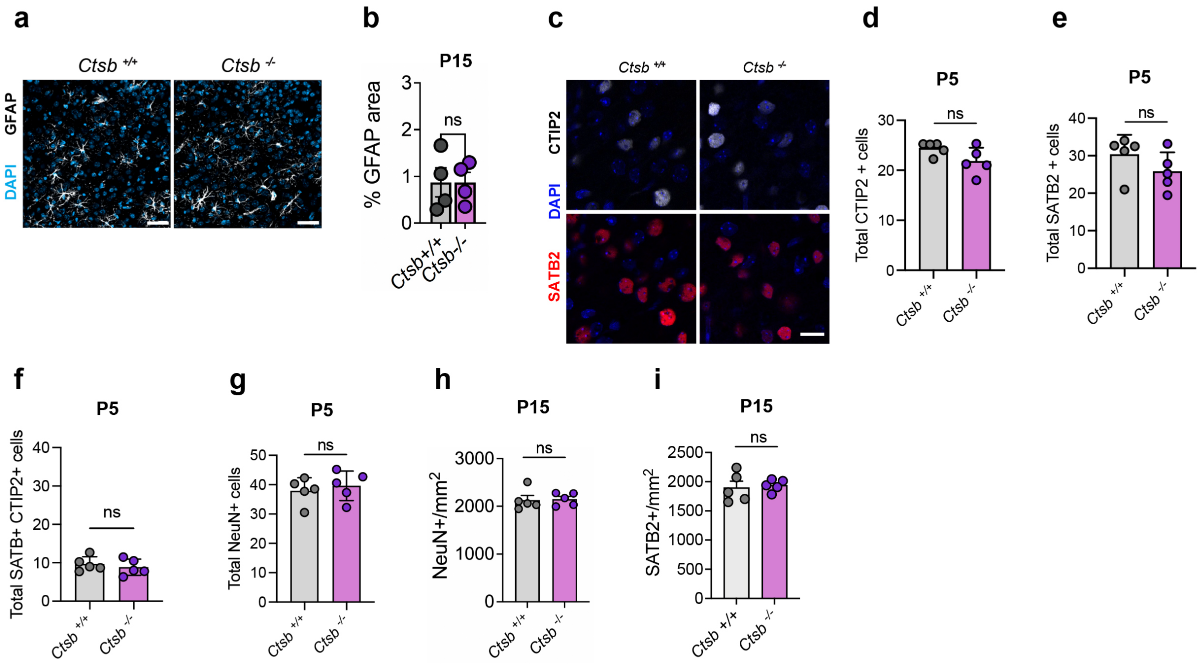

Figure S6: Additional characterization of *Ctsb*-deficient mice, related to Figure 4.

a Representative images of GFAP positive cells (white) in layer 5 of the P15 somatosensory cortex. Left, $Ctsb^{+/+}$ and right, $Ctsb^{-/-}$. Scale bar 40µm.

b Proportion of GFAP positive area across all cortical layers of the P15 somatosensory cortex. Welch's t test. p =ns.

c Representative images of CTIP2 and SATB2 neurons in the somatosensory cortex of P5 *Ctsb* $^{+/+}$ and *Ctsb* $^{-/-}$ mice at P5. Scale = 20 µm.

d $CTIP2^+$ neurons per mm^2 in L5 in *Ctsb* $^{+/+}$ and *Ctsb* $^{-/-}$ mice at P5 (n=5 mice per group). Welch's t-test, ns=0.107.

e $SATB2^+$ neurons density per mm^2 in L5 in *Ctsb* $^{+/+}$ and *Ctsb* $^{-/-}$ mice, P5 (n=5 mice per group). Welch's t-test, ns=0.205.

f $CTIP2^+$ $SATB2^+$ neurons density per mm^2 in L5 in *Ctsb* $^{+/+}$ and *Ctsb* $^{-/-}$ mice, P5 (n=5 mice per group). Welch's t-test, ns.

g NeuN+ neuronal number in L5 in *Ctsb* $^{+/+}$ and *Ctsb* $^{-/-}$ mice at P5 (n=5 mice per group). Welch's t-test, ns = 0.5837.

h Total NeuN + neuronal number in L5 IN *ctsb +/+* and *ctsb -/-* mice at P15 (n= 5 mice per group). Welch's t-test, ns.

i $SATB2^+$ neuron density per mm^2 across all cortical layers in *Ctsb* $^{+/+}$ and *Ctsb* $^{-/-}$ mice at P15 (n=5 mice per group). Welch's t-test, ns =0.7577.

Values were plotted as mean $\pm$ SEM.
