# Supplementary material for: Microglial cathepsin B is necessary for neuronal efferocytosis in zebrafish and mice during brain development
Source: Nat Commun. 2026 Mar 13;17:3881. doi: 10.1038/s41467-026-70350-1 (PMC13125305; doi:10.1038/s41467-026-70350-1)
Supplement: Supplementary file 2 — Description of Additional Supplementary Files [file 41467_2026_70350_MOESM2_ESM.pdf]

Description of Additional Supplementary Files

Movie S1: Control Microglia (GFP) and LysoTracker (Red) real-time acidification event in the optic tectum at 10 dpf. Timestamp (HH:MM). Scale = 5 μm.

Movie S2: *ctsba* CRISPRi KD Microglia (GFP) and LysoTracker (Red) real-time acidification event in the optic tectum at 10 dpf. Timestamp (HH:MM). Scale = 5 μm.
